# Automatic calculation of myocardial external efficiency using a single ^11^C-acetate PET scan

**DOI:** 10.1007/s12350-018-1338-0

**Published:** 2018-06-26

**Authors:** Hendrik J. Harms, Nils Henrik S. Hansson, Tanja Kero, Tomasz Baron, Lars P. Tolbod, Won Y. Kim, Jørgen Frøkiær, Frank A. Flachskampf, Henrik Wiggers, Jens Sörensen

**Affiliations:** 1 Department of Nuclear Medicine, & PET Center, Aarhus University Hospital, Palle Juul-Jensens Boulevard 99, 8200 Aarhus N, Denmark; 2 Department of Cardiology, Aarhus University Hospital, Aarhus, Denmark; 3 Department of Nuclear Medicine & PET, Uppsala University Hospital, Uppsala, Sweden; 4 Department of Medical Sciences, Uppsala Clinical Research Center, Uppsala University, Uppsala, Sweden

**Keywords:** Myocardial efficiency, myocardial energetics, positron emission tomography, ^11^C-acetate

## Abstract

**Background:**

Myocardial external efficiency (MEE) is defined as the ratio of kinetic energy associated with cardiac work [forward cardiac output (FCO)*mean systemic pressure] and the chemical energy from oxygen consumed (MVO_2_) by the left ventricular mass (LVM). We developed a fully automated method for estimating MEE based on a single ^11^C-acetate PET scan without ECG-gating.

**Methods and Results:**

Ten healthy controls, 34 patients with aortic valve stenosis (AVS), and 20 patients with mitral valve regurgitation (MVR) were recruited in a dual-center study. MVO_2_ was calculated using washout of ^11^C -acetate activity. FCO and LVM were calculated automatically using dynamic PET and parametric image formation. FCO and LVM were also obtained using cardiac magnetic resonance (CMR) in all subjects. The correlation between MEE_PET-CMR_ and MEE_PET_ was high (*r* = 0.85, *P* < 0.001) without significant bias. MEE_PET_ was 23.6 ± 4.2% for controls and was lowered in AVS (17.2 ± 4.3%, *P* < 0.001) and in MVR (18.0 ± 5.2%, *P* = 0.004). MEE_PET_ was strongly associated with both NYHA class (*P* < 0.001) and the magnitude of valvular dysfunction (mean aortic gradient: *P* < 0.001, regurgitant fraction: *P* = 0.009).

**Conclusion:**

A single ^11^C-acetate PET yields accurate and automated MEE results on different scanners. MEE might provide an unbiased measurement of the phenotypic response to valvular disease.

**Electronic supplementary material:**

The online version of this article (10.1007/s12350-018-1338-0) contains supplementary material, which is available to authorized users.

## Introduction

A common feature in most cardiomyopathies is a reduction in myocardial external efficiency (MEE)^1^ i.e., an imbalance between cardiac work and total energy consumption by the left ventricle (LV). MEE reflects both mechanical performance and metabolic integrity. A reduction in MEE may result from ischemia, increased wall stress or filling pressures and leads to a further deterioration of LV function. Preserving or restoring MEE is associated with better prognosis and a reduction of symptoms in patients with left ventricular systolic dysfunction,^2^ dilated cardiomyopathy,^3^,^4^ aortic valve stenosis,^5^ and hypertrophic cardiomyopathy^6^ and was thus suggested as a therapeutic target. In addition, MEE may serve as an early marker of cardiac performance and can potentially be used as a more sensitive marker of interventions.^7^,^8^

The gold standard for measuring MEE makes use of pressure-volume loops and the Fick-principle to estimate cardiac work and myocardial oxygen consumption (MVO_2_), respectively. However, the requirement for dual-sided catheterization has ruled out this method in a clinical setting. Accurate non-invasive alternatives are available,^9^ requiring positron emission tomography (PET) with either a combination of ^15^O-labeled PET tracers or, more commonly, ^11^C-acetate washout^10^^–^12 to assess MVO_2_, combined with cardiac magnetic resonance imaging (CMR) or echocardiography to assess Cardiac Output (CO) and LV mass (LVM).

Since MEE integrates measurements of LV mass, function, and oxidative metabolism, a near-simultaneous acquisition of all measurements is preferred. This could be accomplished using hybrid PET/CMR or ECG-gated PET,^13^ but neither can be applied to all potential patient groups and both require significant post-processing of data, which would introduce observer bias. An optimal solution would be to extract CO and LVM directly from the dynamic PET data in an automated and scanner-independent fashion. Recently, automated methods of obtaining forward cardiac output (FCO)^14^ and LVM^15^ have become available, using only a dynamic ^11^C-acetate PET scan. Therefore, the aim of this study was to evaluate the accuracy of MEE derived from a single dynamic ^11^C-acetate PET scan. In addition, obtained MEE values for patients with aortic valve stenosis (AVS) and mitral valve regurgitation (MVR) are compared with those of healthy controls.

## Materials and Methods

### Patient Population

This study consists of a retrospective analysis of three groups of subjects undergoing efficiency measures in research studies from two different research sites. The first group consisted of 34 patients (69.0 ± 8.4y, 24 men) with AVS and varying degrees of heart failure (22 asymptomatic, 8 NYHA class II and 4 NYHA class III patients). All patients had sinus rhythm, no signs of myocardial ischemia and aortic valve area ≤ 1.2 cm^2^ and/or transaortic maximal velocity of 3.0–5.0 m s^−1^ based on echocardiography. The second group consisted of 20 patients (56.4 ± 15.6y, 19 men) with significant mitral regurgitation (regurgitant fraction > 30% on echocardiography) and with no or mild symptoms of heart failure (15 and five NYHA class I and II, respectively). The final group consisted of 10 healthy controls (62.5 ± 4.4y, 7 men) with no signs or history of cardiac disease. AVS patients and controls were scanned at the Aarhus University Hospital, Aarhus, Denmark whilst MVR patients were scanned at the Uppsala University Hospital, Uppsala, Sweden. The study was approved by the respective local ethical committees and all subjects gave written informed consent prior to inclusion in this study.

### Image Acquisition

#### PET

^11^C-acetate synthesis was done according to Pike^16^ with minor in-house modifications. After a fasting period of > 4 h, AVS patients and controls underwent ^11^C-acetate PET scan on a Siemens Biograph TruePoint TrueV 64 PET/CT scanner. Following a scout CT scan, a low-dose CT scan (120 kV, 30 mAs) was performed. After this, a 27-minute list mode emission scan was performed, starting simultaneously with automated injection of 407 ± 30 MBq ^11^C-acetate as a 5–10 mL bolus (1 mL·s^−1^) in a peripheral vein, followed by a 35-mL saline flush (2.0 mL·s^−1^). List mode emission data were rebinned into a dynamic series consisting of 29 time frames using all data. Dynamic images were reconstructed using the TrueX algorithm, applying all appropriate corrections as supplied by the vendor.

MVR patients were scanned on a GE discovery ST with an acquisition protocol identical to that of AVS patients and controls. Data were reconstructed using the 3D IR algorithm with all appropriate corrections as supplied by the vendor.

#### CMR

AVS patients and controls were scanned on an Ingenia 1.5 T whole body scanner (Philips Healthcare, Best, The Netherlands) as described in^14^ and.^15^ MVR patients were scanned on an Ingenia 3 T whole body scanner (Philips Healthcare, Best, The Netherlands) with an 80 mT·m^−1^ gradient system, using similar imaging protocols. Details on exact settings can be found in the supplemental files.

### Calculation of Myocardial External Efficiency

Myocardial external efficiency (MEE) was calculated using the methods as described in^9^,^17^ and the workflow used in this study is summarized in the supplemental file. MEE was defined as

1 $$ {\text{MEE}} = \frac{{\text{EW}}}{{\text{TE}}} = \frac{{{\text{MAP}} \cdot {\text{FCO}} \cdot 1.33 \cdot 10^{ - 4} }}{{{\text{MVO}}_{2} \cdot {\text{LVM}} \cdot 20}} \cdot 100\% $$

In which EW is the effective external work performed by the heart (J); TE is the total energy use (J); MAP is the mean arterial pressure (mmHg); FCO is the forward cardiac output (mL·min^−1^); MVO_2_ is the myocardial oxygen consumption (mL·g^−1^·min^−1^); LVM is the mass of the left ventricle (LV, g), and 1.33 · 10^−4^ and 20 are the conversion factors from 1 mmHg mL to J and from 1 mL of O_2_ to J, respectively. The numerator, output energy (*E*_out_) is similar to cardiac work, expressed in Joules, and represents the area enclosed within a pressure-volume loop. The denominator represents input energy (*E*_in_), the total energy consumed by the LV.

HR and MAP were measured 1 minute before and 1 and 5 minutes after injection and averaged for calculation of MEE. MVO_2_ was derived from ^11^C-acetate PET data, whereas both FSV and LVM were derived from either CMR (FSV_CMR_ and LVM_CMR_) or ^11^C-acetate PET data (FSV_PET_ and LVM_PET_). Supplemental Figure 1 summarizes the steps required for calculation of MEE, which are outlined below. An example of a dynamic time-series and all relevant intermediate steps of the analysis for each of the patient categories is shown in Supplemental Figures 2, 3, and 4.

#### MVO_2_

PET scans were analyzed using aQuant^18^ (available at no cost for collaborative, non-commercial research purposes via https://aquantsoft.com/go/aquantresearch). Arterial (*C*_A_(*t*)) and right-ventricular (*C*_RV_(*t*)) blood concentrations were obtained automatically using cluster analysis.^14^,^18^ Arterial blood concentrations were converted into arterial plasma input functions (*C*_P_(*t*)) by applying the average plasma metabolite correction as presented by Sun et al.^19^ Finally, *C*_P_(*t*) was used for calculation of washout rate *k*_2_ using a standard single compartment model^20^ with all appropriate corrections for spillover from the blood and blood volume fractions:

2 $$ C_{\text{PET}} \left( t \right) = \left( {1 - V_{\text{A}} } \right) \cdot {K_1} \cdot C_{\text{P}} \left( t \right) \otimes e^{{ - k_{2} \cdot t}} + V_{\text{A}} \cdot C_{\text{A}} (t) + V_{\text{V}} \cdot C_{\text{V}} (t) $$

In which *C*_PET_(*t*) represents the myocardial time-activity curve, and *C*_P_(*t*), *C*_A_(*t*), and *C*_RV_(*t*) are the aforementioned blood time-activity curves. *V*_A_ represents arterial blood volume fraction (dimensionless), *K*_1_ the uptake rate of ^11^C-acetate in tissue (mL·g^−1^·min^−1^), *k*_2_ the washout rate of ^11^C-acetate (in min^−1^), *V*_RV_ is the right-ventricular spillover fraction (dimensionless).

Parametric images were generated using basis function methods^18^ and used for automatic segmentation of the LV, as described in detail elsewhere.^15^ In brief, for each short-axis slice the ventricular mid-point is defined using the center of gravity of V_A_, from which radial profiles are generated every 10°. Then, for each profile, the first and last point above 2/3rd of the maximum of each profile were considered to represent the endo- and epicardial borders. This process was repeated for each profile and each short-axis slice until the entire LV was segmented.

After LV segmentation, activity concentrations in the LV were extracted and used as *C*_PET_(*t*) in Eq. (2), yielding average *k*_2_ of the entire LV. This global *k*_2_ was converted into MVO_2_ using the empirically derived conversion factors of Sun et al.^19^:

3 $$ {\text{MVO}}_{2} = 1.35 \cdot k_{2} - 9.6 \cdot 10^{ - 3} $$

Identical values of MVO_2_ were used for both MEE_PET-CMR_ and MEE_PET_.

#### Forward cardiac output

For AVS patients and controls, CMR-based forward stroke volume (FSV_CMR_) was calculated from phase contrast velocity measurement in the LV outflow tract. Flow analysis was performed using the freely available software Segment (version 1.9 R3746).^21^ As AVS regularly results in turbulent flow patterns, phase contrast velocity was imaged at the level of the LV outflow tract where flow velocity was laminar. For MVR patients, FSV_CMR_ was calculated from phase contrast velocity measurement in the ascending aorta and flow analyses were performed on a ViewForum workstation (Philips, Best, the Netherlands). FSV_CMR_ was multiplied with HR during PET to obtain FCO

FSV based on PET (FSV_PET_) was calculated using indicator-dilution techniques using the methods described in 14 correcting for scanner-dependent differences between FSV_PET-CMR_ and FSV_PET_ as presented in that study. In brief, the peak of the first-pass of the ^11^C-acetate bolus through the arterial blood, obtained for calculation of MVO_2_ as described above, was isolated automatically from *C*_A_(*t*). Using this peak, forward cardiac output was estimated using

4 $$ {\text{FCO}}_{\text{PET}} = \frac{I}{{\mathop \smallint \nolimits C_{\text{A}} \left( t \right)}} $$

In which FCO_PET_ is forward cardiac output (mL·min^−1^); *I* is the injected dose of ^11^C-acetate (Bq) and *∫C*_A_(*t*) is the area under the curve of the isolated peak (Bq·mL^−1^·min).

#### LVM

LVM_CMR_ was derived by manually tracing the endo- and epicardium in end-diastole on short-axis cine images tracing using the software Segment v1.9 R2854^21^ for AVS patients and ViewForum (Philips) for MVR patients. LVM_PET_ was defined using the volume of interest of the LV used to obtain *C*_PET_(*t*). For both LVM_PET-CMR_ and LVM_PET_, a density of myocardial tissue of 1.05 g cm^3^ was assumed.

### Statistical Analysis

Data are presented as mean ± SD. Correlation and agreement were assessed using linear regression and Bland Altman plots. Paired *t* tests were used to evaluate systematic differences. Repeatability coefficient (RPC) was defined as 2 times the standard deviation of the difference. Differences between patient groups were assessed using One-way ANOVA followed by student’s *t* tests. The independent capacity of MEE_PET_ and MEE_PET-CMR_ for prediction of NYHA class was assessed by ordinal logistic fitting.

## Results

Data of one AVS patient showed significant motion during PET, and this patient was excluded from further analysis.

### Patient Characteristics

Table 1 shows relevant hemodynamic parameters and all parameters used in calculation of MEE_PET_ for all three groups. Compared to controls, systolic blood pressure was significantly higher for AVS (*P* = 0.015) but not for MVR (*P* = 0.09). Neither MAP (*P* = 0.55 and 0.30) nor FCI (*P* = 0.27 and 0.17) were significantly different from controls, resulting in similar values for EW (*P* = 0.47 and 0.56 for AVS and MVR). MVO_2_ in patients did not differ from that of controls (*P*=0.26 and 0.92 for AVS and MVR) but LVM was larger (*P* = 0.003 and < 0.001), resulting in a significantly increased TE (*P* = 0.003 and 0.019).

**Table 1:** Mean ± standard deviation of all hemodynamic or PET-derived parameters involved in calculation of myocardial external efficiency (MEE) for healthy controls, aortic valve stenosis (AVS), and mitral regurgitation (MVR) patients

	Controls (*n* = 10)	AVS (*n* = 33)	MVR (*n* = 20)
SBP (mmHg)	124 ± 11	139 ± 18*	134 ± 15
DBP (mmHg)	79 ± 6	79 ± 11	71 ± 13^†^
HR (min^−1^)	62 ± 8	65 ± 11	60 ± 12
MAP (mmHg)	97 ± 9	99 ± 12	92 ± 12^†^
MVO_2_ (mL_O2_·g^−1^·min^−1^)	0.10 ± 0.02	0.12 ± 0.04	0.11 ± 0.03
FCI (L·min^−1^·m^−2^)	2.4 ± 0.4	2.6 ± 0.6	2.6 ± 0.4
LVMI (g·m^−2^)	63.8 ± 8.4	89.3 ± 25.0**	90.5 ± 17.2***
EW (J)	60.9 ± 15.0	66.2 ± 21.0	64.3 ± 14.6
TE (J)	260 ± 57	399 ± 137**	385 ± 152*
MEE (%)	23.6 ± 4.2	17.2 ± 4.3***	18.0 ± 5.2**

*SBP*, systolic blood pressure; *DBP*, diastolic blood pressure; *HR*, heart rate; *MAP*, mean arterial pressure; *MVO*_*2*_, rate of oxygen consumption; *FCI*, forward cardiac index; *LVMI*, left ventricular mass corrected for body-surface area; *EW*, external work; *TE*, total energy usage

*, ** and ***P < 0.05, < 0.01 and < 0.001 vs healthy controls

^†^*P* < 0.05 vs AVS

### Validation vs CMR

**Figure 1:**
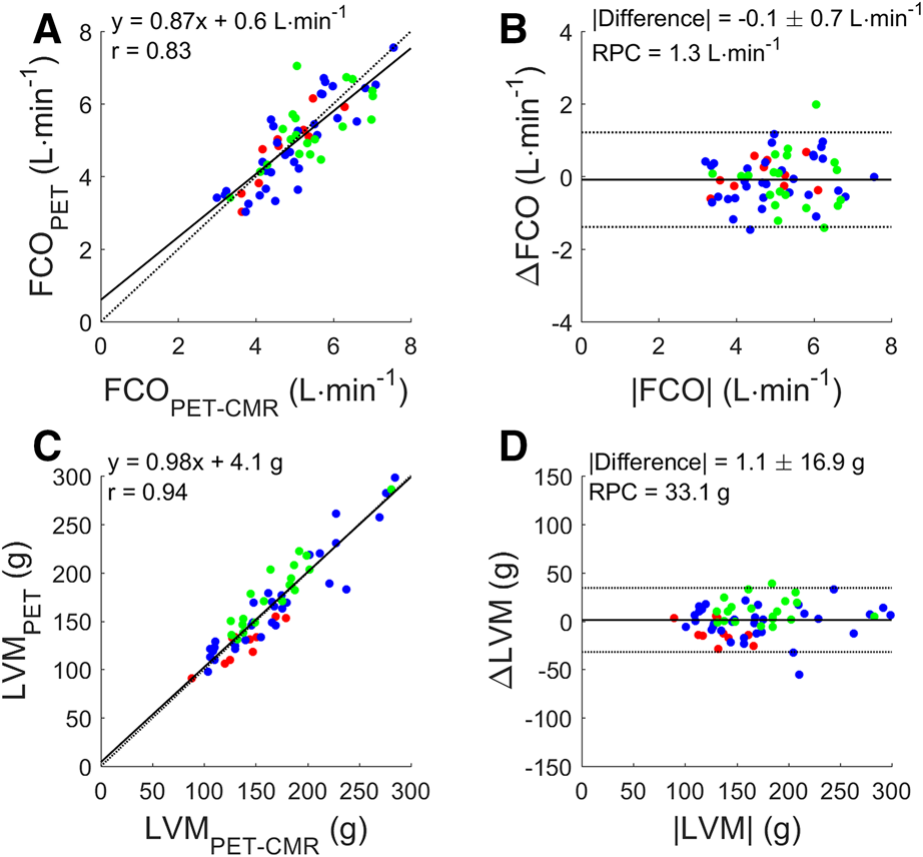
Correlation (**A**, **C**) and Bland Altman plot (**B**, **D**) of forward cardiac output (**A**, **B**) and left ventricular mass (**C**, **D**) based on CMR (FCO_PET-CMR_ and LVM_PET-CMR_) and PET (FCO_PET_ and LVM_PET_). Black and gray lines indicate the line of identity and the linear fit in (**A**, **C**) and the mean difference and the 95% confidence interval in (**B**, **D**). RPC: repeatability coefficient. Red: healthy controls (*n* = 10), blue: AVS patients (*n* = 33), green: MVR patients (*n* = 20)

**Figure 2:**
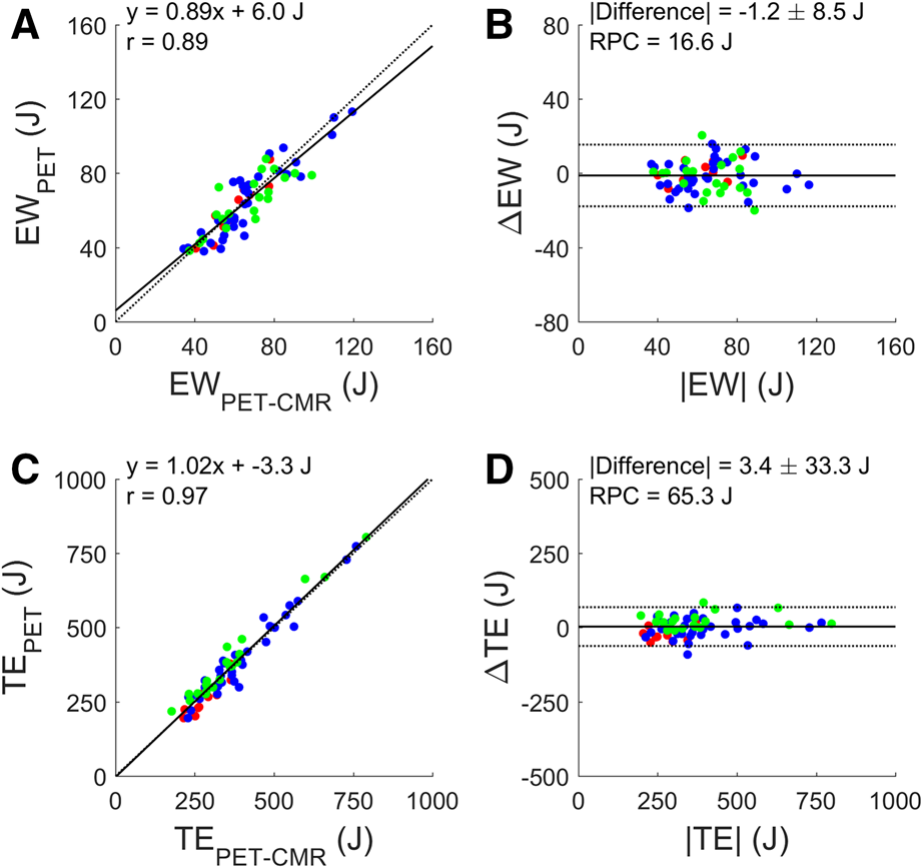
Correlation (**A**, **C**) and Bland Altman plot (**B**, **D**) of external work (**A**, **B**) and total energy use (**C**, **D**) based on CMR (EW_PET-CMR_ and TE_PET-CMR_) and PET (EW_PET_ and TE_PET_). Black and gray lines indicate the line of identity and the linear fit in (**A**, **C**) and the mean difference and the 95% confidence interval in (**B**, **D**). *RPC*, repeatability coefficient. Red: healthy controls (*n* = 10), blue: AVS patients (*n* = 33), green: MVR patients (*n* = 20)

**Figure 3:**
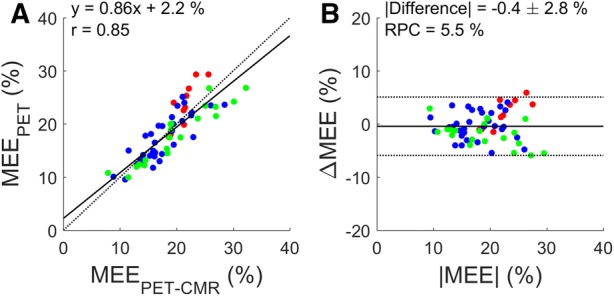
Correlation (**A**) and Bland Altman plot (**B**) of myocardial external efficiency based on a combined PET-CMR protocol (MEE_PET-CMR_) and PET-only (MEE_PET_). Black and gray lines indicate the line of identity and the linear fit in (**A**) and the mean difference and the 95% confidence interval in (**B**). RPC: repeatability coefficient. Red: healthy controls (*n* = 10), blue: AVS patients (*n* = 33), green: MVR patients (*n* = 20)

Correlation between PET and CMR was good for both FCO and LVM (*r* = 0.83 and *r* = 0.94, respectively, Figure 1). Correlation between PET- and CMR-derived measures was high for both EW and TE (*r* = 0.89, and 0.97, respectively, Figure 2). Bland Altman analysis revealed no significant differences for EW (− 1.2 ± 8.5 J, *P* = 0.445) or TE (3.4 ± 33.3 J, *P* = 0.564). Finally, correlation of MEE_PET_ and MEE_PET-CMR_ was high (*r* = 0.85, Figure 3) without significant bias (absolute difference of − 0.4 ± 2.8%, *P* = 0.511).

EW_PET_ and EW_PET-CMR_ were not significantly different for any patient group. On the other hand, TE_PET_ was significantly higher than TE_PET-CMR_ for MVR (22.9 ± 26.0 J, *P* = 0.001) and significantly lower for controls (− 28.1 ± 19.0 J, *P* = 0.001). Finally, MEE_PET_ was significantly different as compared to MEE_PET-CMR_ for controls (2.8 ± 3.0%, *P* = 0.02) and MVR (− 1.8 ± 2.3%, *P* = 0.011) but not for AVS (− 0.2 ± 2.6%, *P* = 0.62). Residual analysis identified the difference in FCO as the main source of difference in MEE in all groups (Controls: *r* = 0.58, *P* = 0.02; AVS: *r* = 0.79, *P* < 0.001; MVR: *r* = 0.58, *P* = 0.01).

### Clinical Characteristics of MEE

**Figure 4:**
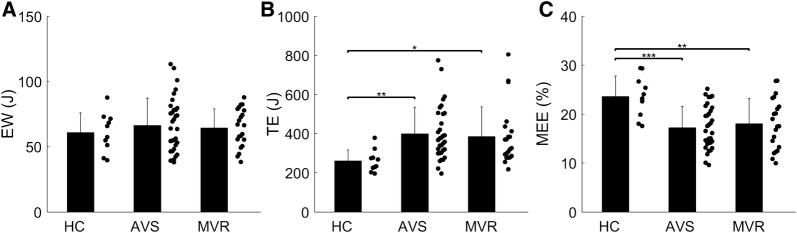
Mean ± SD and individual values for EW_PET_ (**A**), TE_PET_ (**B**) and MEE_PET_ (**C**) for all three groups. *, **, *** indicate significance level of < 0.05, < 0.01, and < 0.001, respectively

**Figure 5:**
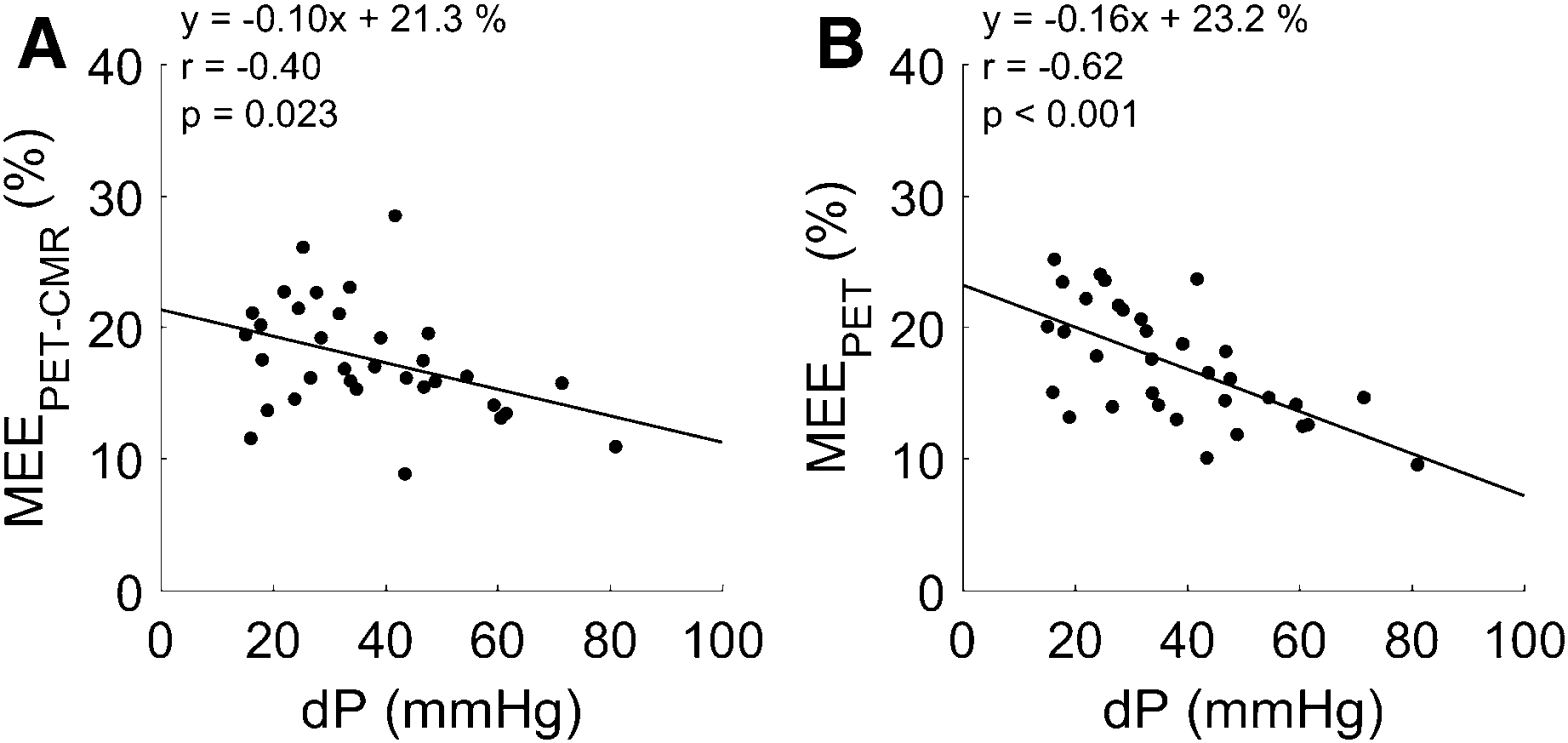
Correlation of mean pressure gradient over de aortic valve and MEE derived using PET-CMR (**A**) and PET-only (**B**) for AVS patients

**Figure 6:**
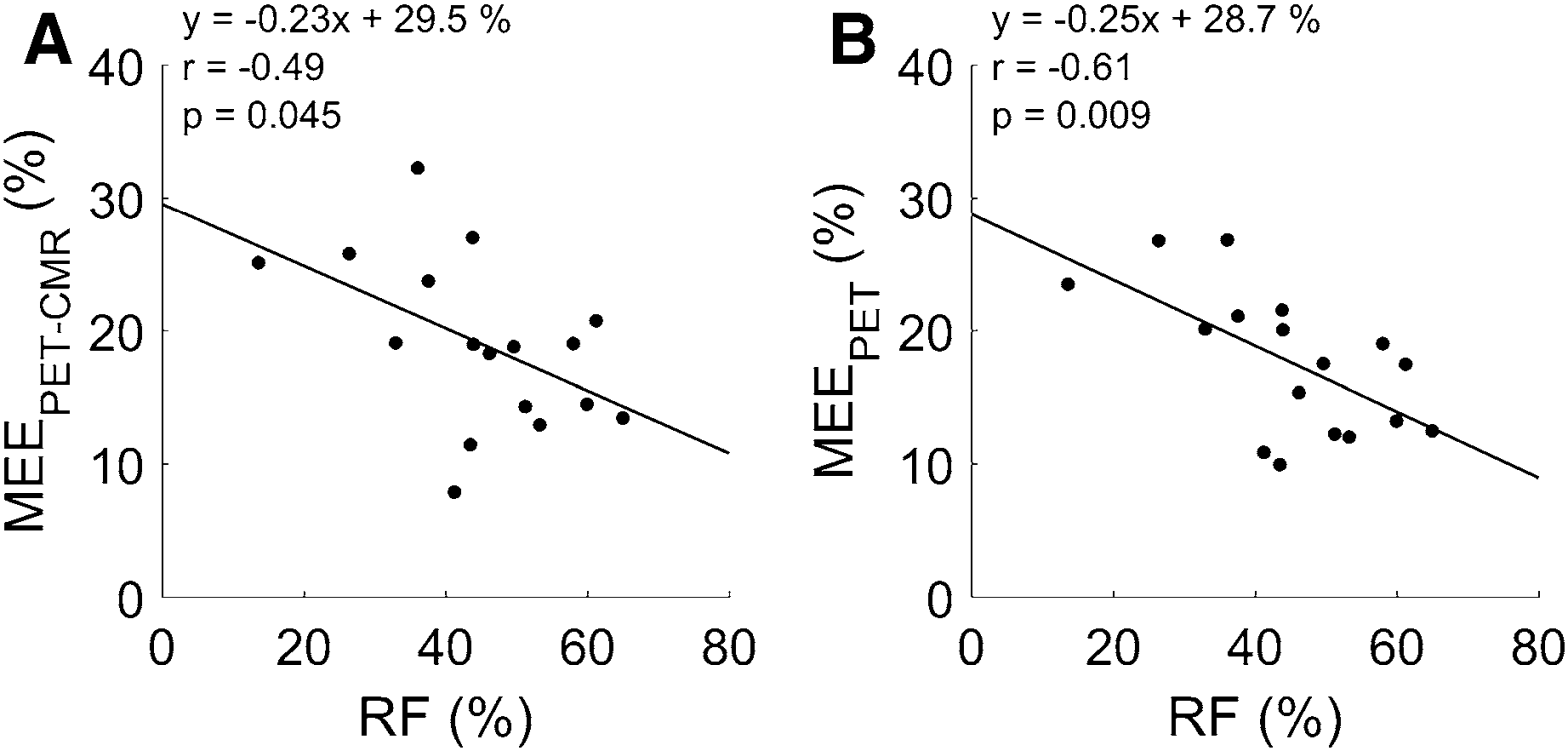
Correlation of CMR-derived regurgitant fractions and MEE derived using PET-CMR (**A**) and PET-only (**B**) for MVR patients. *RF*, regurgitant fraction

**Figure 7:**
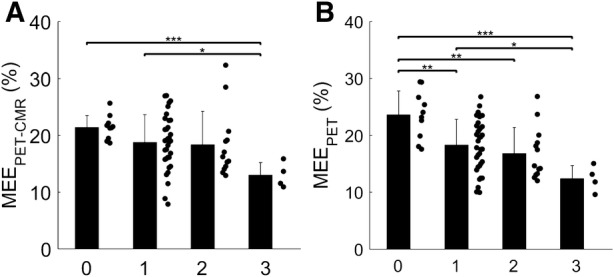
Mean ± standard deviation of MEE_PET-CMR_ (**A**) and MEE_PET_ (**B**) per NYHA heart failure class. *, **, and *** denote a *P* value below 0.05, 0.01, and 0.001, respectively

When comparing patient groups, MEE_PET_ was significantly lower for both AVS (*P* < 0.001) and MVR (*P* = 0.006) as compared to controls (Figure 4), whilst MEE_PET-CMR_ was significantly lower for AVS (*P* = 0.009) but not MVR (*P* = 0.49). Mean aortic gradient in AVS was correlated to MEE_PET-CMR_ (*r* = − 0.40, *P* = 0.023) and to MEE_PET_ (*r* = − 0.62, *P* < 0.001, Figure 5), of which the correlation to MEE_PET_ was significantly higher (*P* = 0.01). In addition, regurgitant fraction was correlated to both MEE_PET_ (*r* = − 0.61, *P* = 0.009) and to MEE_PET-CMR_ (*r* = − 0.49, *P* = 0.045, Figure 6) with no significant difference between correlations (*P* = 0.10). Finally, MEE_PET_ was strongly associated with NYHA class (ANOVA *P* < 0.001) and significantly separated most groups (Figure 7), while the association was less clear for MEE_PET-CMR_ (ANOVA *P* = 0.03). Using Ordinal Logistic fitting only MEE_PET_ was independently associated with NYHA class (*χ*^2^ 14.2, *P* < 0.0002), compared to MEE_PET-CMR_ (*χ*^2^ 3.8, *P* = 0.052).

## Discussion

This study presents a fully automated method of calculating myocardial external efficiency (MEE) solely from a dynamic ^11^C-acetate PET/CT scan without the use of ECG-gating. This method eliminates the need for separate measurements of cardiac output and LV mass, reducing protocol duration, cost and analysis time. MEE is a sensitive marker of cardiac performance and has been used as surrogate end-point in several interventional studies,^7^,^8^ potentially lowering the sample size as compared to traditional markers such as ejection fraction or outcomes.

Both FCO and LVM have been validated vs CMR before^14^,^15^ in a subset of the subjects included in this study. LVM measured by PET without ECG-gating in particular appears to perform well across different scanners, compared to CMR. FCO requires a scanner-dependent correction factor, as previously observed.^14^ The difference in FCO was identified as the only significant source of MEE deviation between modalities. Since associations towards clinical parameters were stronger for MEE_PET_, the difference in FCO points to a significant change in LV loading conditions between the CMR and PET scans. This is encouraging in terms of using MEE from PET alone as an end-point in multicenter trials, but including other scanner models requires further validation against CMR to establish correction factors.

In a test-retest study, we show that repeatability of MEE_PET-CMR_ and MEE_PET_ were high and not significantly different (coefficient of variation of 6.3% and 9.5%, respectively, *P* = 0.25).^22^ The range of control values was narrower for MEE_PET-CMR_ as compared to MEE_PET_, as can also be appreciated in Figure 7, suggesting a higher sensitivity and the need for smaller groups when using MEE_PET-CMR_ as marker for efficiency. However, Figure 7 also shows that the difference between controls and patients with valvular diseases was higher for MEE_PET_, suggesting that the (non-significantly) increased test-retest variability for MEE_PET_ is largely off-set by an increased effect size. The exact benefit of using either MEE_PET_ and MEE_PET-CMR_ must be studied in larger clinical studies. Finally, the use of a combined PET-echocardiography protocol was not recommended from a reproducibility point of view.^22^

MEE calculated with both approaches resulted in control values ranging from 17 to 30%, which is in line with previously published invasive measurements.^9^ Patients were consecutively recruited from on-going larger studies and two-thirds were asymptomatic. Half of the patients had MEE lower than any control subject, suggesting that attenuated efficiency at rest is common in valvular diseases. MEE_PET_ was significantly correlated to the echocardiographically derived mean pressure gradient of the aortic valve in AVS patients (*r* = − 0.62, *P* < 0.001) and to the regurgitant fraction obtained from CMR in MVR patients (*r* = − 0.61, *P* = 0.009). In addition, MEE corresponded to the subjective level of disease burden defined by the NYHA class. This indicates that MEE reflects the phenotypic response to the causative disease process in valvular disease. To what extent MEE can be used to define the optimal time point for valvuloplasty requires larger outcome studies, for which the current study suggests that a PET-alone approach might perform better than serial multimodality imaging.

Care has to be taken when comparing different values for MEE in literature. In some studies, pressure gradient over the aortic valve is used in Eq. (1) (replacing MAP by MAP+∆P), which is likely to minimize differences in MEE. Similarly, when the total stroke volume including the blood that regurgitates over either valve is used instead of FSV, differences between controls and MVR are expected to be smaller and it becomes clear that there are conceptual differences between MEE obtained in either case. MEE as calculated using Eq. (1) represents the energetic cost of the entire LV required to pump a certain amount of blood into the systemic circulation, ignoring any pathological pressures in the LV cavity and excluding any regurgitating volume. This could be considered the net efficiency of the whole heart as a pump or *global LV efficiency* and reflects both the metabolic and mechanical state of the heart. The result of this study suggests that global LV efficiency is a sensitive marker of generic cardiac performance. If, on the other hand, regurgitation or elevated LV pressures are taken into account, MEE represents the energetic cost of displacing blood in any direction which can be considered the efficiency of the cardiomyocytes, reflecting the metabolic state of the heart specifically i.e., the *metabolic efficiency*. Noteworthy, global LV and metabolic efficiency deviate only in the case of valvular dysfunction. When pressure gradients are essential, echocardiography can be performed during a PET examination,^23^ although echocardiography is limited by the acoustic window and the presence of significant operator differences. Similarly, total stroke volume can be obtained using gated PET but accuracy of that method is so far suboptimal.^13^ However, the present study shows that it is feasible to obtain MEE according to either definition during a single scan session.

This study has several limitations that need to be acknowledged. The PET acquisition protocols were aligned between both participating sites, but since differences between PET scanners were observed widespread implementation of this all-in-one approach in multicenter studies requires further validation. CMR equipment and protocols differed between sites, which is likely to induce bias.

Secondly, this study mainly included subjects with valvular abnormalities which typically show discrepancies in pressure-volume loops. The assumption that EW, formally defined as the area encompassed in a patient’s pressure-volume loop, can be approximated by the product of MAP and SV is often incorrect in these patients.^9^ Errors in EW estimates can be reduced by utilizing forward instead of total stroke volume and/or adding the mean pressure gradient over the aortic valve, although the latter increases complexity of the method. As discussed above, care has to be taken when considering the use of mean pressure gradients or whether to use the forward or total stroke volume. In this study, we chose to use forward stroke volume and exclude mean pressure gradient to obtain a ‘net’ or global LV efficiency, equally affected by mechanical abnormalities of the heart and valves and by any potential metabolic alterations.

To conclude, myocardial efficiency can be measured accurately using a single ^11^C-acetate PET/CT scan, without the need for additional imaging modalities. Because of the more generic, highly automated, and less logistically demanding approach, this novel technique might widen the applicability of MEE to more patient groups.

## New Knowledge Gained

The work presented in this study enables a simplified, faster, and more automated assessment of myocardial external efficiency using a single ^11^C-acetate scan. Using a single scan protocol instead of a combined PET-CMR protocol leads to lower potential errors due to differences in loading conditions. When applying this method to a cohort of controls and patients with valvular diseases, MEE based on PET-only correlated more closely to the underlying disease state and to NYHA class.

## Electronic supplementary material

Below is the link to the electronic supplementary material.

Supplementary material 1 (DOCX 2271 kb)

## Abbreviations

MEE: Myocardial external efficiency
TE: Total energy use
EW: External work
LVM: Left ventricular mass
FCO: Forward cardiac output
MVO_2_: Myocardial oxygen consumption
CMR: Cardiac magnetic resonance imaging
PET: Positron emission tomography
AVS: Aortic valve stenosis
MVR: Mitral valve regurgitation
