# Supplementary material for: Automatic calculation of myocardial external efficiency using a single 11C-acetate PET scan
Source: J Nucl Cardiol. 2018 Jun 26;25(6):1937–44. doi: 10.1007/s12350-018-1338-0 (PMC6280778; doi:10.1007/s12350-018-1338-0)
Supplement: Supplementary file 1 — Supplementary material 1 (DOCX 2271 kb) [file 12350_2018_1338_MOESM1_ESM.docx]

**Supplemental files**

**CMR scanning protocol**

AS patients and HC were scanned on an Ingenia 1.5T whole body scanner (Philips Healthcare, Best, The Netherlands). A survey scan was performed followed by an ECG-triggered temporally resolved cine using a balanced steady-state free precession sequence during breath hold. A slice thickness of 8 mm, slice gap of 0 mm, repetition time/echo time/flip angle of 3.1 ms/1.55 ms/60°, respectively, 350 x 350 mm field of view, 352 x 352 acquisition matrix, and 30 phases within one cardiac cycle were used. A stack of 16 to 18 LV short-axis slices were acquired covering the entire LV. Directly after this, breath-hold through-plane phase-contrast CMR acquisitions were performed at the level of the left-ventricular outflow tract (LVOT) using an echo and repetition time of 2.5 and 4.1 ms, respectively, a phase percentage of 60%, field of view (FOV) of 350 mm, a matrix size of 140 by 140 with 8mm slice thickness and 25 phases and a single excitation. To avoid aliasing, encoding velocity was set to 100-200 cm/s based on Pulse Wave Doppler imaging from echocardiography performed just prior to CMR.

MI patients were scanned on an Ingenia 3T whole body scanner (Philips Healthcare, Best, The Netherlands) with an 80 mT/m gradient system, a 32 channel cardiac coil in supine position and retrospectively gated vector ECG for cardiac triggering. Functional images were obtained with a single-shot steady state free precession (SSFP) cine sequence covering the left ventricular myocardium from apex to base in 6-mm thick short-axis slices with 4 mm gaps. Parameters used were: TR shortest (3.4ms), TE shortest (1.7ms), flip angle 45°, bandwidth 1243 Hz/pixel, 30 phases/cardiac cycle, field-of-view 320 mm and matrix 160´ 154. Following this, based on orthogonal balanced turbo field echo images, respiratory triggered through-plane phase-contrast MI acquisitions were obtained at the level of the aorta ascendens during free breathing. Typical parameters were as follows: echo time = 2.7 ms, repetition time = 4.7 ms, phase percentage = 81%, field of view = 320 mm, matrix = 128 x 104, number of phases = 40, number of excitations = 2, slice thickness = 8 mm, encoding velocity = 100-200 kollas.

**Supplemental Figure 1**. Flowchart of calculation of MEE_PET-CMR_ (A) and MEE_PET_ (B), showing analysis steps for ^11^C-acetate PET (blue) and CMR (red). For both MEE_PET-CMR_ and MEE_PET_, washout rate k_2_ of ^11^C-acetate is obtained in order to obtain MVO_2_. First, the arterial input function is obtained using cluster analysis(1), followed by generation of parametric images which are then used to segment the left-ventricular (LV) wall automatically. The LV time-activity curve is then obtained and, using the obtained arterial input function, fitted to a single-tissue compartment model to obtain washout rate k_2_. k_2_ is then converted into MVO_2_ using the linear conversion as described in (2). After obtaining MVO_2_, for MEE_PET-CMR_, separate assessments of forward stroke volume (FSV) and LV mass (LVM) are used. For MEE_PET_ on the other hand, FSV is obtained from the arterial input function using indicator-dilution analysis(3) while LVM is obtained from the size of the segmented LV used to extract the LV activity curve(4).


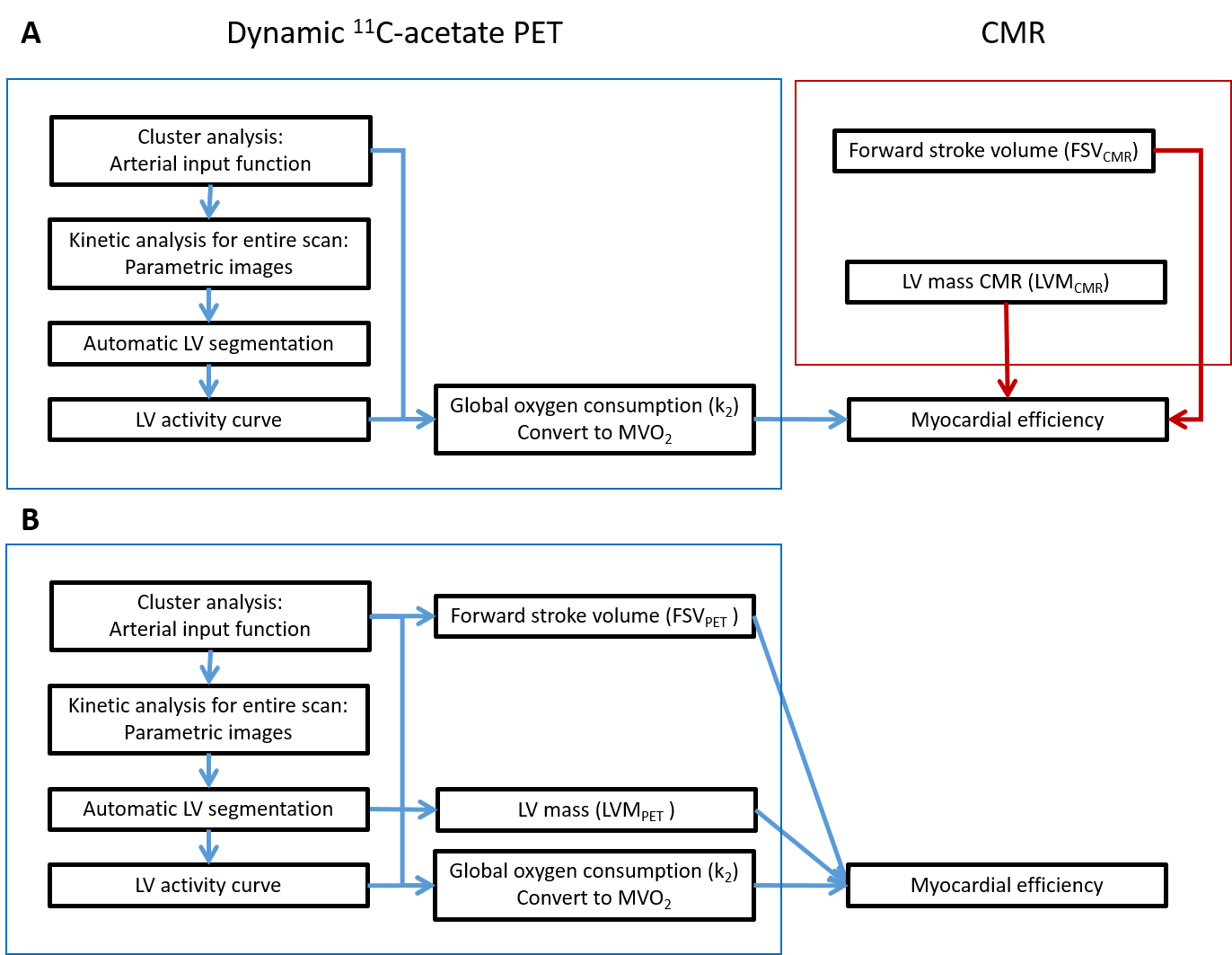


**Supplemental figure 2.** Example of an analysis in a healthy control. Top: maximum-intensity projection of each of the 29 frames of the dynamic study. Middle row: example of a segmented arterial (red) and venous (blue) cluster (left) and their corresponding time-activity curves. The first-pass peak of the arterial cluster is then isolated and used to calculate forward cardiac output. Bottom row: example of a short-axis image with contours projected on them (left). This process is repeated for all included short-axis images and the volume between the contours for all slices is used for m_LV_. For the volume between the contours, the average time-activity curve is obtained and fitted using a single tissue compartment model, obtaining washout rate k_2_. This washout rate, combined with the mean arterial pressure, FCO and m_LV_ is then used to calculate MEE.


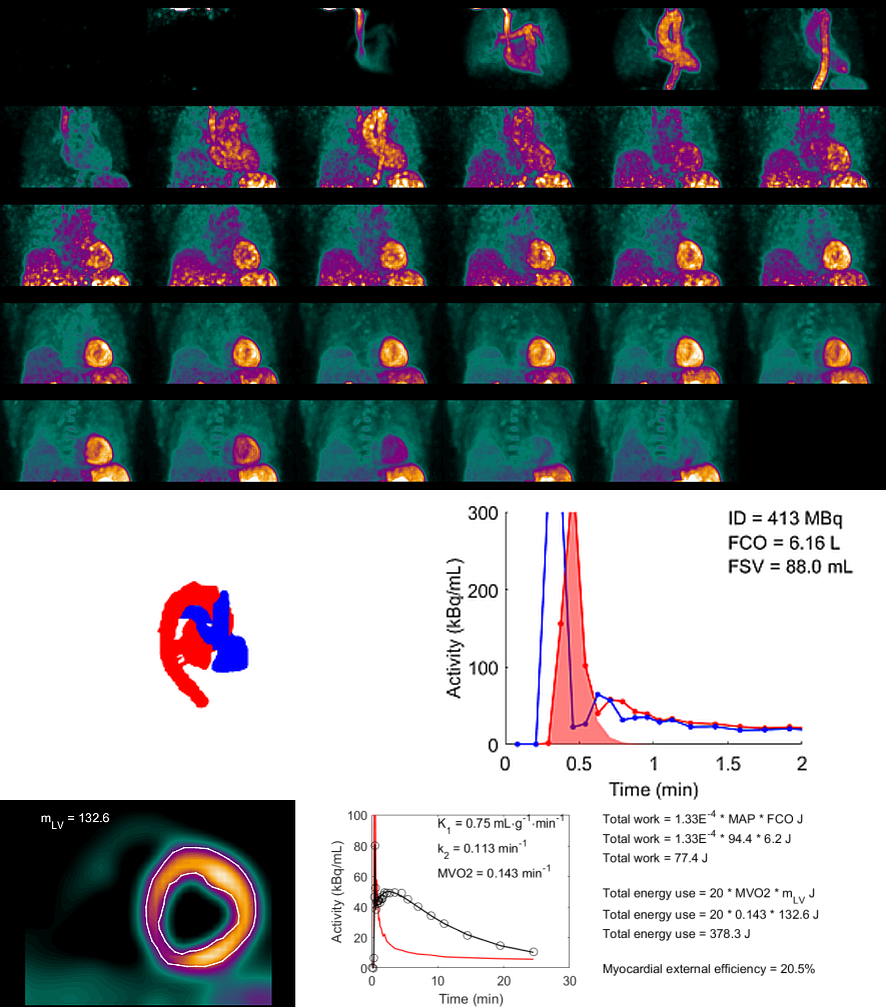


**Supplemental figure 3.** Example of an analysis in a patient with aortic valve stenosis. Displayed images are similar as in supplemental figure 2.


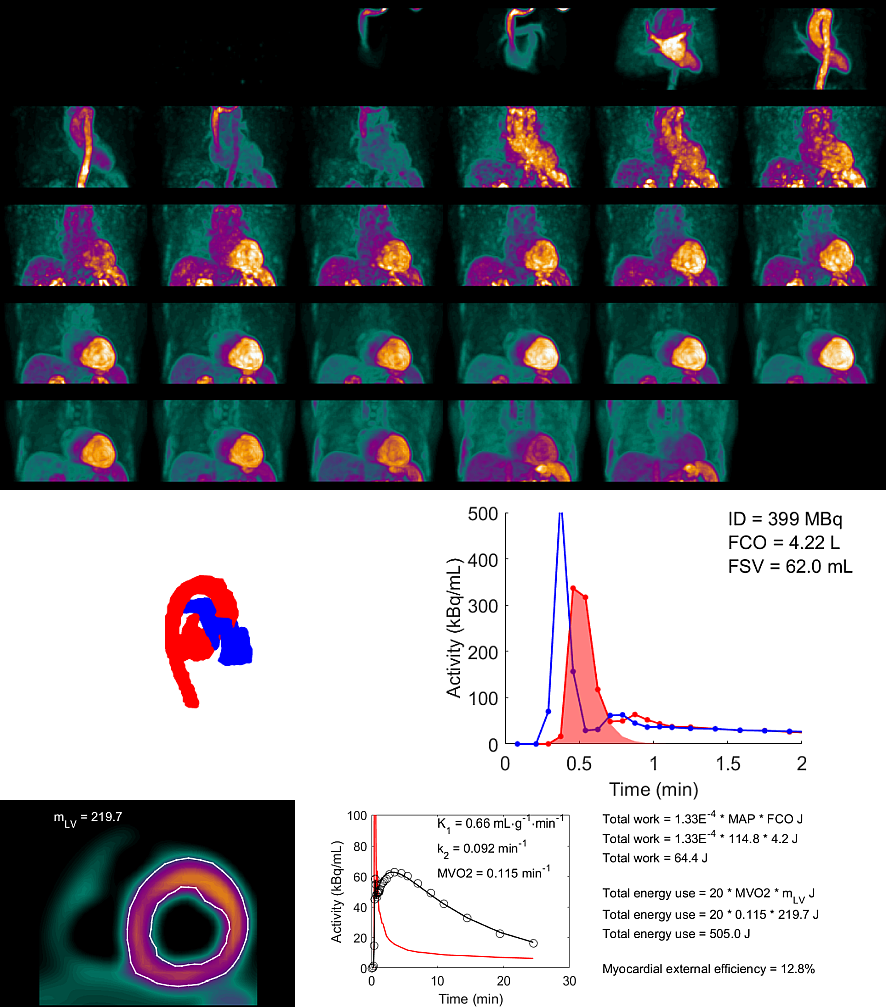


**Supplemental figure 4.** Example of an analysis in a patient with mitral valvular regurgitation. Displayed images are similar as in supplemental figure 2.


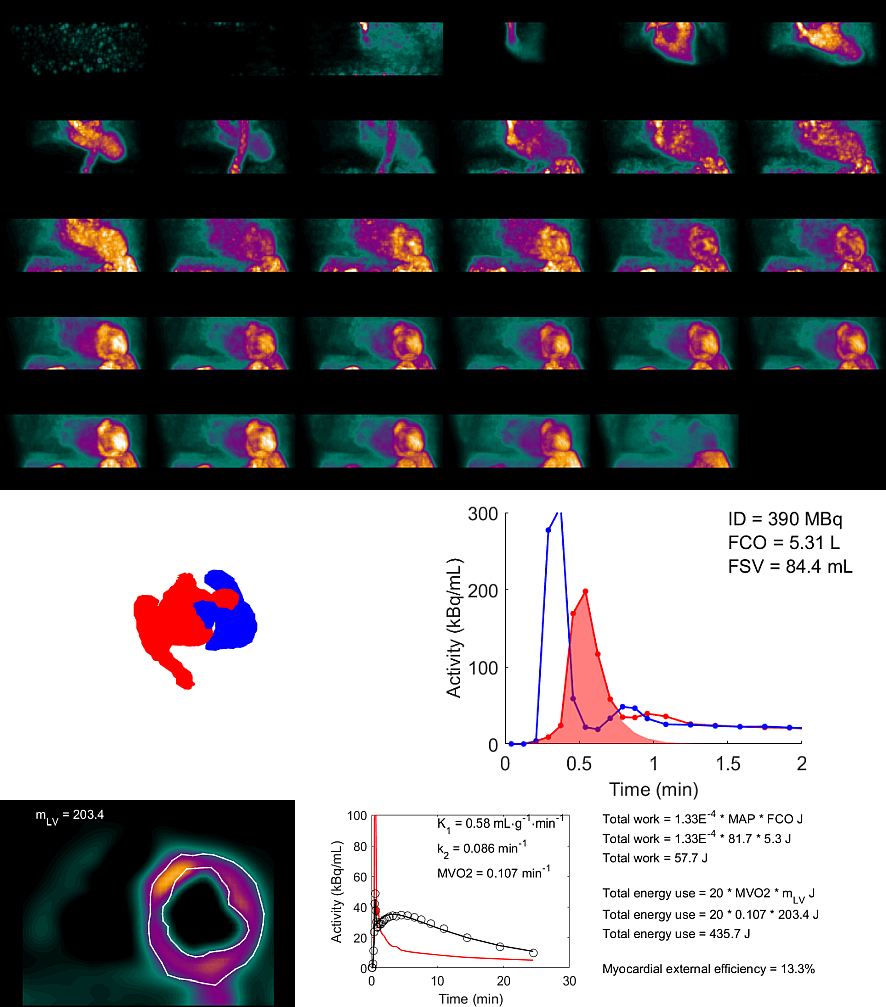


Reference List

(1) Harms HJ, Knaapen P, de HS, Halbmeijer R, Lammertsma AA, Lubberink M. Automatic generation of absolute myocardial blood flow images using [15O]H2O and a clinical PET/CT scanner. *Eur J Nucl Med Mol Imaging*. 2011;38:930-939.

(2) Sun KT, Yeatman LA, Buxton DB, et al. Simultaneous measurement of myocardial oxygen consumption and blood flow using [1-carbon-11]acetate. *J Nucl Med*. 1998;39:272-280.

(3) Harms HJ, Tolbod LP, Hansson NH, et al. Automatic extraction of forward stroke volume using dynamic PET/CT: a dual-tracer and dual-scanner validation in patients with heart valve disease. *EJNMMI Phys*. 2015;2:25.

(4) Harms HJ, Hansson NH, Tolbod LP, et al. Automatic extraction of myocardial mass and volumes using parametric images from dynamic non-gated PET. *J Nucl Med*. 2016.
